# Case Report: Multifocal non-invasive follicular thyroid neoplasm with papillary-like nuclear features presenting in a female child

**DOI:** 10.12688/f1000research.23687.2

**Published:** 2020-09-21

**Authors:** Asmaa Gaber Abdou, Hayam Aiad, Nancy Asaad

**Affiliations:** 1 Department of Pathology, Menoufia University, Shebein Elkom, Menoufia, 32511, Egypt

**Keywords:** NIFTP, children, multifocality

## Abstract

Non-invasive follicular thyroid neoplasm with papillary-like nuclear features (NIFTP) was introduced as a separate entity by the World Health Organization in 2017 with strict inclusion and exclusion criteria.  Most NIFTP cases have been reported in adults and few cases have been diagnosed in children. Here, we present a classic case of NIFTP affecting a 10-year old female child. We also review previous reports of NIFTP in children regarding size, focality, nodal metastasis, recurrence, type of operation and follow-up data. The present report adds a new case of NIFTP in the paediatric age group characterized by multifocality, absence of nodal invasion and indolent course until last follow-up, recommending less aggressive management.

## Introduction

Generally, the diagnosis of papillary thyroid carcinoma (PTC) has increased over the past several decades ^1^, partly due to increased recognition of the follicular variant of PTC ^2^. The subjectivity in diagnosis of this variant and the indolent behaviour of encapsulated or non-invasive forms, led to revision and follow-up of a large number of these cases by international multidisciplinary collaborative group ^3, 4^. Consequently, the encapsulated variant of PTC was reclassified as non-invasive follicular thyroid neoplasm with papillary-like nuclear features (NIFTP), which had strict inclusion and exclusion criteria for this diagnosis. The term NIFTP was then introduced as a separate entity by the World Health Organization in 2017, with a category of follicular tumour of uncertain malignant potential and well-differentiated tumour of uncertain malignant potential ^5^. The majority of NIFTP reports have been in adults. Here, we present a classic case of NIFTP affecting a 10-year old female child.

## Case report

A female patient of 10 years presented to our department with an enlarged thyroid that had been observed by her mother. No previous relevant family history was recorded.

**Figure 1.**
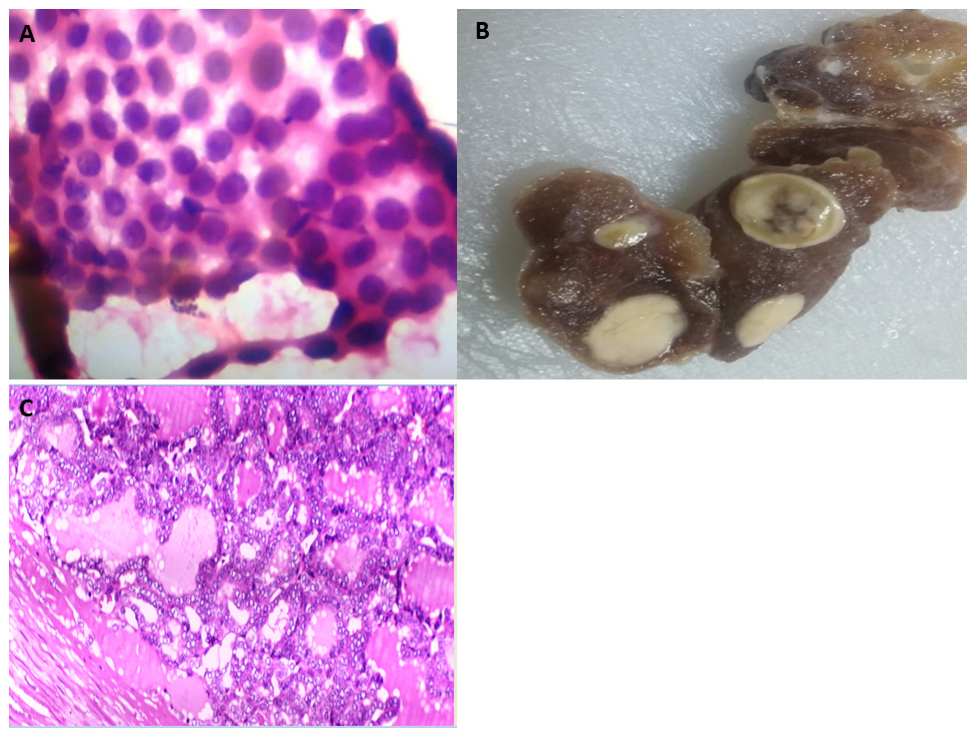
Right thyroid lobe results from patient. ( **A**) Cytologic features of fine needle aspiration cytology showing cohesive sheet of follicular epithelial cells, including some which were rounded and others that were elongated with occasional grooved nuclei (hematoxylin and eosin, mag. ×600). ( **B**) Gross picture of affected right lobe after total thyroidectomy showing two well circumscribed whitish nodules. ( **C**) Histopathological examination of nodule of resected thyroid revealing a capsulated nodule formed of microfollicles lined by follicular epithelial cells, which had enlarged pale crowded nuclei together with nuclear grooves and inclusions (nuclear features of papillary thyroid carcinoma)(hematoxylin and eosin, mag. ×400).

Ultrasound revealed two suspicious nodules on the right side of the thyroid lobe. No pathological lymph node enlargement was reported. Ultrasound guided fine needle aspiration cytology was performed and the results showed sheets of follicular epithelial cells, some were elongated with occasional nuclear grooves and inclusions ( Figure 1A). This was diagnosed as atypical thyroid lesion indefinite for malignancy (THY3a).

The patient was submitted for total thyroidectomy within one month from her first presentation. On resection, the right thyroid lobe measured 5.5 × 3.5 × 3 cm with two well-defined, firm, grayish white nodules. One nodule measured 2 × 1.5 cm and the other measured 1.5 × 1.5 cm ( Figure 1B). The left lobe and isthmus measured 4.5 × 3 cm and 1 × 0.5 cm, respectively. 

Histological examination of the two nodules resected from the right thyroid lobe revealed well-circumscribed capsulated nodules formed of microfollicles, lined by follicular epithelial cells with wide-spread nuclear features of papillary thyroid carcinoma ( Figure 1C). There was no evidence of capsular or vascular invasion, true papillae, trabeculae or solid arrangement. The patient did not receive any specific medications before surgery and she was followed up for 12 months with no evidence of recurrence or nodal involvement.

## Discussion

Most NIFTP cases have been previously reported in adults and data concerning this diagnosis in children is scarce; only 21 cases in children have been reported in the English literature within the last two years ( Table 1) ^6– 10^. Preoperative diagnosis of our case was based on ultrasound data and the cytology was not obviously malignant. The cytologic smears of NIFTP were usually hypercellular showing follicular epithelial cells arranged in microfollicles without papillae formation and they showed subtle features of papillary thyroid carcinoma but with infrequent or absent nuclear inclusions. NIFTP cytology was commonly interpreted as follicular lesion of undetermined significance in 30% (categories III and IV according to Bethesda system), follicular neoplasm in 21%, suspicious for malignancy in 24%, malignant in 8%, bnign in 10% and non-diagnostic in 3% ^11, 12^. Although the above findings would suggest lobectomy, our patient was submitted for total thyroidectomy and as has been done in previously reported cases ^6, 7, 9, 10^.

**Table 1.** Characteristics of reported non-invasive follicular thyroid neoplasm with papillary-like nuclear features in children.

	Age (years)	Gender F:M	Size (cm)	Focality	Recurrence	Metastasis	Operation	Follow up (months)
Wang *et al.*, 2019 (3 cases) ^6^	16–17	2:1	0.4–3.1	Single	No	No	Total thyroidectomy	**15–138**
Rosario and Mourão, 2018 (4 cases) ^7^	9-15	3:1	1.7-2.4	Single	No	No	Total thyroidectomy	**24-108**
Rossi *et al.*, 2018 (2cases) ^8^	<19	1:1	<2 > 2	Single	No	No	NA	**84**
Mariani *et al.*, 2018 (10 cases) ^9^	14.4	3.5:1	2.1	7 cases single 3 cases multifocal	No	2 cases with lymph node metastases	Total thyroidectomy	**NA**
Samuels *et al.*, 2018 (2 cases) ^10^	14	2:1	1.1-4.5	NA	No	No	Total thyroidectomy	**NA**
The current case	10	Female	1.5-2	Multifocal	No	No	Total thyroidectomy	**12**

F:M, female to male ratio, NA: not available

On a molecular level, NIFTP shares follicular neoplasm in RAS mutations but it lacks *BRAF ^V600E^* mutations, which is a common event in papillary thyroid carcinoma ^13^. Immunohistochemistry for *BRAF ^V600E^* mutations is available on paraffin blocks. Nuclear pseudinclusions are important diagnostic criteria for PTC, which could be highlighted by CK19 immunostaining in comparison to routine hematoxylin and eosin ^14^. The latter authors demonstrated absence of CK19 positive nuclear pseudoinclusions in the investigated 7 cases of NIFTP.

The current report demonstrated a classic case of NIFTP affecting a young female child, agreeing with previous reports that there are more cases in women than men ( Table 1). Although not common, multifocality has been reported previously for NIFTP in adults ^15^ and in children ^9^. The size of NIFTP lesion is usually small, rarely exceeding 2 cm in diameter ( Table 1). 

More aggressive therapy is recommended for PTC in childhood and adolescence ^16^ but the indolent behaviour reported for NIFTP necessitates less aggressive management in children, as well as adults. Therefore, completion lobectomy is not recommended for postoperative cases diagnosed as NIFTP ^8^. NIFTP in children has a similar outcome as cases reported in adults, suggesting that paediatric NIFTP behaves indolently, as evidenced by the absence of local recurrence and nodal metastasis ^6^.

The present report adds a new case of NIFTP in the paediatric age group characterized by multifocality, absence of nodal invasion and indolent course - until last follow-up, recommending less aggressive management of this disease.

## Consent

Written informed consent was obtained from the patient's father for the publication of this case report and any associated images.

## Data availability

All data underlying the results are available as part of the article and no additional source data are required.
