# Supplementary material for: Case Report: Multifocal non-invasive follicular thyroid neoplasm with papillary-like nuclear features presenting in a female child
Source: F1000Res. 2020 Sep 21;9:645. Originally published 2020 Jun 25. [Version 2] doi: 10.12688/f1000research.23687.2 (PMC7594245; doi:10.12688/f1000research.23687.2)
Supplement: Peer review history

# Peer review: Reviewer response for version 2

**DOI:** 10.5256/f1000research.29612.r69816

**Published:** 2020-10-28

**Authors:** Osama Sharaf Eldin

**Affiliations:** 1 Weston General Hospital, Weston-super-Mare, UK

The case is well written and with full explanation of the diagnostic category. The introduction included the accurate classification of the entity and why the nomenclature changed.The figures and tables are well presented. The references are up to date.

 No further comments and I agree for this case report to be indexed without changes.

Are enough details provided of any physical examination and diagnostic tests, treatment given and outcomes?

Yes

Is the case presented with sufficient detail to be useful for other practitioners?

Yes

Is sufficient discussion included of the importance of the findings and their relevance to future understanding of disease processes, diagnosis or treatment?

Yes

Is the background of the case’s history and progression described in sufficient detail?

Yes

Reviewer Expertise:

Molecular Pathology, Digital Pathology and experimental pathology

I confirm that I have read this submission and believe that I have an appropriate level of expertise to confirm that it is of an acceptable scientific standard.

# Peer review: Reviewer response for version 1

**DOI:** 10.5256/f1000research.26135.r70314

**Published:** 2020-09-08

**Authors:** Hanan Alshenawy

**Affiliations:** 1 Pathology Department, Faculty of Medicine, Tanta University, Tanta, Egypt

This is a case report about a case of NIFTP in thyroid.

- The case is well clinically presented with full clinical data.
- The procedure as FNAC is also presented well with clear figure.
- Histopathology is shortly presented with clear good figure.
- The discussion should be in more details.
- Is there any role for immunohistochemistry?

Are enough details provided of any physical examination and diagnostic tests, treatment given and outcomes?

Yes

Is the case presented with sufficient detail to be useful for other practitioners?

Yes

Is sufficient discussion included of the importance of the findings and their relevance to future understanding of disease processes, diagnosis or treatment?

Partly

Is the background of the case’s history and progression described in sufficient detail?

Yes

Reviewer Expertise:

histopathology

I confirm that I have read this submission and believe that I have an appropriate level of expertise to confirm that it is of an acceptable scientific standard.
